# Serum-Derived microRNAs as Prognostic Biomarkers in Osteosarcoma: A Meta-Analysis

**DOI:** 10.3389/fgene.2020.00789

**Published:** 2020-08-11

**Authors:** Huan Luo, Peng Wang, Hua Ye, Jianxiang Shi, Liping Dai, Xiao Wang, Chunhua Song, Jianying Zhang, Jitian Li

**Affiliations:** ^1^College of Public Health, Zhengzhou University, Zhengzhou, China; ^2^Henan Key Laboratory of Tumor Epidemiology, Zhengzhou University, Zhengzhou, China; ^3^Zhengzhou University, Henan Academy of Medical and Pharmaceutical Sciences, Zhengzhou, China; ^4^Laboratory of Molecular Biology, Henan Luoyang Orthopedic Hospital (Henan Provincial Orthopedic Hospital), Zhengzhou, China

**Keywords:** miRNA, osteosarcoma, prognosis, serum, biomarker, meta-analysis

## Abstract

Recent reports suggest that microRNAs (miRNAs) may serve as prognostic biomarkers in osteosarcoma. Due to osteosarcoma's early metastasis and poor prognosis, it is very important to find novel prognostic biomarkers for improving osteosarcoma's prognosis. Herein we propose a meta-analysis for serum miRNA's prognostic value in osteosarcoma. In this study, the literature available from PubMed, Web of Science, Embase, and Cochrane Library databases was reviewed. The pooled hazard ratios (HRs) with their 95% confidence intervals (CIs) were calculated to evaluate miRNAs prognostic values. A total of 20 studies investigating serum miRNAs were included in this meta-analysis; the initial terminal point of these reports included overall survival (OS), progression-free survival (PFS), disease-free survival (DFS), and recurrence-free survival (RFS). For prognostic meta-analyses, the pooled HR for terminal events of higher expression of miRNAs and lower expression of miRNAs were 5.68 (95% CI 4.73–6.82, *P* < 0.05) and 3.78 (95% CI 3.27–4.37, *P* < 0.05), respectively. Additionally, subgroup analyses were conducted based on the analysis methods applied and clinicopathological features reported. In the pooled analyses, the miRNA expression levels are associated with poor prognosis according to both univariate and multivariate analyses. Furthermore, serum miRNAs (miRNA-195, miRNA-27a, miRNA-191, miRNA-300, miRNA-326, miRNA-497, miRNA-95-3p, miRNA-223, miRNA-491-5p, miRNA-124, miRNA-101, miRNA-139-5p, miRNA-194) were associated with poor OS and found to be closely correlated with clinical stage and distant metastasis in osteosarcoma. The results illustrate that low or high expression of these specific miRNAs are both potentially useful as prognostic serum biomarkers in osteosarcoma, and miRNAs (miRNA-195, miRNA-27a, miRNA-191, miRNA-300, miRNA-326, miRNA-497, miRNA-95-3p, miRNA-223, miRNA-491-5p, miRNA-124, miRNA-101, miRNA-139-5p, miRNA-194) may indicate clinical stage and metastasis in this form of cancer.

## Introduction

Patient survival in osteosarcoma has improved in recent decades. Osteosarcoma is the most common malignant bone tumor, with a worldwide incidence of approximately one to three cases annually per million (Kansara et al., 2014). Current therapies include surgical resection and combination neoadjuvant chemotherapy, which is reported to have a curative effect in ~70% of patients (Collins et al., 2013). Metastasis and recurrence are common challenges in refractory osteosarcoma, that worsen patient prognosis (Bielack et al., 2002). The highly malignant nature of osteosarcoma, as well as its high rates of recurrence and lung metastasis represent strong concerns (Jones et al., 2012; Ogawa et al., 2013). Clinically, histological examination of the biopsy specimens is preferred for the diagnosis or prognostic evaluation of osteosarcoma. However, such invasive tests may be burdensome when monitoring the progression of the disease, and the accuracy of diagnosis and prognostic evaluation may vary because of differences in sample collection and personnel. Therefore, it is essential to develop novel approaches for the timely diagnosis of osteosarcoma in order to achieve better prognosis (Gu et al., 2014).

MiRNAs are small (about 21-nucleotide-long) non-coding RNAs which can regulate gene expression (Filipowicz et al., 2008). Elevated or downregulated miRNAs may act as oncogenes or tumor suppressors in various cancers (Hayashita et al., 2005; He et al., 2005; Kent and Mendell, 2006; Tian et al., 2019). Additionally, miRNAs that are stable in serum or plasma, or in other biological samples, may have potential utility as diagnostic or prognostic biomarkers in different cancers (Calin and Croce, 2006; Esquela-Kerscher and Slack, 2006; Mitchell et al., 2008; Zhou et al., 2016). These findings show that miRNAs warrant attention as potential novel biomarkers for diagnosis or prognosis in osteosarcoma.

Although numerous recent studies have reported a correlation between prognosis in osteosarcoma and miRNA expression, none have demonstrated sufficient evidence for clinical translation of their findings. For instance, two previous meta-analyses have concluded the prognostic value of miRNA expression in osteosarcoma (Cheng et al., 2017; Kim et al., 2017); however, in these studies, either tissue or both tissue and blood were used as samples. Tissue samples' obtainment are invasive for patients than serum samples. To optimally obtain samples from patients and increase patients' acceptability, it is important for us to find novel serum biomarker for osteosarcoma. To the best of our knowledge, very few studies have provided robust evidence on the potential prognostic utility of serum miRNAs in osteosarcoma. Therefore, in the present work, we conducted a meta-analysis of studies in which serum samples were analyzed, to explore the prognostic value of miRNAs in osteosarcoma. Following which, subgroup analyses included analysis method and clinicopathological features were also explored to better analyze the prognostic value of various groups.

## Methods

This study was implemented according to the guidelines of the Meta-analysis of Observational Studies in Epidemiology (MOOSE) (Stroup et al., 2000), and the Preferred Reporting Items for Systematic Reviews and Meta-Analysis (PRISMA) guidelines (Moher et al., 2009). We have completed the prognostic value of serum microRNA. In constructing the prognostic value of serum microRNA, we comply with the population, interventions, comparators, out-comes, and study designs (PICOS) principle to complete the research design.

### Selection of Studies

The literature available in PubMed, Web of Science, Embase, and Cochrane Library databases, up to June 20, 2020, was investigated. The combination of search terms used was (osteosarcoma OR osteogenic sarcoma) AND (microRNA OR miRNA OR miR) AND (prognosis OR survival OR prognostic OR outcome). Only studies of the Chinese population published in English were included, and studies analyzing samples other than serum were excluded.

### Inclusion and Exclusion Criteria

Inclusion criteria for studies in this review were as follows: (1) studies investigating the utility of miRNAs for evaluating prognosis in osteosarcoma, (2) serum miRNAs' assay method based on quantitative real-time polymerase chain reaction, (3) studies presenting sufficient data to allow calculation of HR and 95% CI, and (4) studies in which a cut-off value was defined. Studies were subject to the following exclusion criteria: (1) studies reporting duplicate data; studies in non-Chinese populations, (2) non-English publications, review articles, or meta-analysis, (3) studies reporting insufficient data for pooled analysis, and (4) studies of tissue, cell lines, or animal experiments.

### Quality Assessment and Data Extraction

For prognostic meta-analyses, the quality of included studies was assessed using the Newcastle–Ottawa Scale (NOS), based on the following categories: selection, comparability, and outcome; the highest score was 9, with scores ≥6 indicating studies of high quality (Stang, 2010). The extracted data and information included were as follows: the first author, the year of publication, the country of origin, osteosarcoma sample size, sample type, cut off value, miRNAs characteristics, analysis methods, clinical outcomes, and detection methods. Two investigators retrieved and assessed the literature, respectively, and disagreements were resolved by extensive discussion.

### Statistical Methods

All analyses were performed using STATA 12.0 software. Based on the information provided in the included studies, the pooled HRs with 95% CIs were calculated using this meta-analysis model. Forest plots were used to estimate the effect of miRNA expression on overall survival (OS), progression-free survival (PFS), disease-free survival (DFS), and recurrence free-survival (RFS) (Hong et al., 2014; Zhang et al., 2014a,b; Cai et al., 2015; Tang et al., 2015; Wang N. G. et al., 2015; Wang T. et al., 2015; Yang et al., 2015; Cao et al., 2016; Dong et al., 2016; Liu et al., 2016; Niu et al., 2016; Pang et al., 2016; Wang S. N. et al., 2017; Wang Z. et al., 2017; Cong et al., 2018; Li et al., 2018; Yao et al., 2018; Zhou et al., 2018; Shi et al., 2020). *I*^2^ index was used to assess the between-study heterogeneity, with *I*^2^ > 50% indicating a large degree of heterogeneity; in this case, a random effect model was applied. *I*^2^ ≤ 50% implied that there was no significant heterogeneity, and the fixed effect model was used. Next, subgroup analyses were conducted to identify potential sources of heterogeneity and assess the prognostic value of different subgroups; the level of significance was set at *P* < 0.05. In addition, Begg's test (Begg and Mazumdar, 1994) and Egger's test (Egger et al., 1997) were performed to assess the publication bias; values of *P* < 0.05 indicated significant publication bias.

## Results

### Characteristics of the Included Studies and Quality Assessment

**Figure 1:**
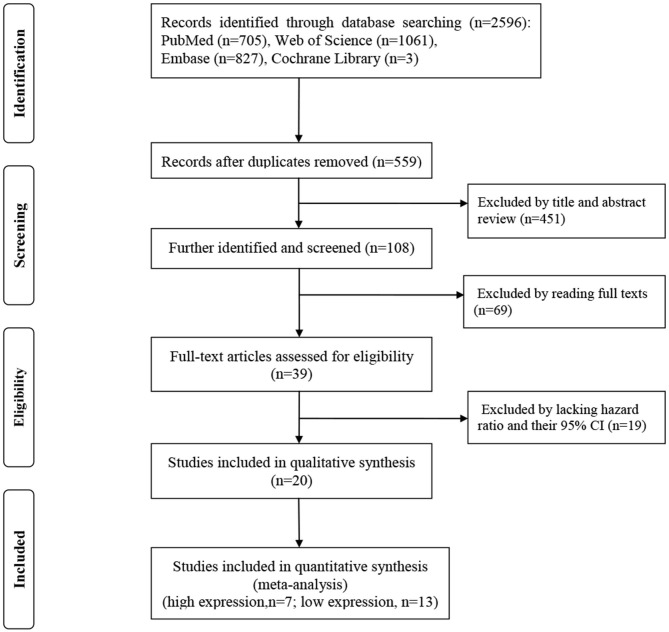
Flow-process diagram of the study selection process.

The screening process for the studies is shown in detail in Figure 1. A primary search of the PubMed, Web of Science, Embase, and Cochrane Library databases, using the search strategy described, identified 2,596 articles. The innovative contribution of this work is focused on studies which using serum sample, herein studies using plasma or tissue were excluded, as such, all included studies examined serum samples. The data extracted from the included studies, the quality of the reports and heterogeneity are shown in Tables 1, 2. The osteosarcoma sample size ranged from 60 to 185 subjects. The assay method was based on qRT-PCR. Cut off values were defined in the included studies to differentiate between high-expression miRNAs and low-expression miRNAs, and multivariate or univariate analyses were performed. The quality of each study was high according to the NOS (Stang, 2010). A total of 20 studies and 2,242 osteosarcoma patients were included in this prognostic meta-analysis.

**Table 1:** The extracted data and quality assessment of literature on the prognostic utility of miRNAs in osteosarcoma.

**Author/miRNA**	**Year**	**Country**	**Sample size**	**Sample type**	**Cut off value**	**miRNA expression with poor prognosis**	**Assay method**	**Analysis method**	**Outcome**	**NOS score**
Hong miRNA-29a/29b	2014	China	80	Serum	2.85/3.27	High	qRT-PCR	M	OS, DFS	8
Zhang miRNA-196a/196b	2014	China	100	Serum	4.86/5.48	High	qRT-PCR	M	OS, DFS	8
Cai miRNA-195	2014	China	166	Serum	1.44	Low	qRT-PCR	M	OS, DFS	8
Zhang miRNA-133b/206	2014	China	100	Serum	2.66/2.84	Low	qRT-PCR	M	OS, DFS	8
Yang miRNA-221	2015	China	108	Serum	2.42	High	qRT-PCR	M	RFS, OS	7
Wang miRNA-152	2015	China	80	Serum	NR	Low	qRT-PCR	M	OS	6
Tang miRNA-27a	2015	China	166	Serum	3.70	High	qRT-PCR	M	OS, DFS	8
Wang miRNA-191	2015	China	100	Serum	3.56	High	qRT-PCR	M	OS, DFS	7
Dong miRNA-223	2016	China	112	Serum	1.21	Low	qRT-PCR	M	OS	6
Liu miRNA-300	2016	China	114	Serum	NR	High	qRT-PCR	U/M	OS/DFS	6
Cao miRNA-326	2016	China	60	Serum	Mean level	Low	qRT-PCR	M	OS	6
Niu miRNA-95-3p	2016	China	133	Serum	0.75	Low	qRT-PCR	M	OS	7
Pang miRNA-497	2016	China	185	Serum	4.80	Low	qRT-PCR	U/M	OS	7
Li miRNA-542-3p	2017	China	76	Serum	0.87	High	qRT-PCR	U/M	OS, PFS	7
Wang miRNA-491	2017	China	102	Serum	Mean level	Low	qRT-PCR	U/M	OS	7
Wang miRNA-491-5p	2017	China	72	Serum	NR	Low	qRT-PCR	M	OS, DFS	6
Cong miRNA-124	2017	China	114	Serum	0.37-fold	Low	qRT-PCR	M	OS, DFS	6
Yao miRNA-101	2018	China	152	Serum	Median level	Low	qRT-PCR	U/M	OS, RFS	6
Zhou miRNA-139-5p	2018	China	98	Serum	Median level	Low	qRT-PCR	U/M	OS	7
Shi miRNA-194	2020	China	124	Serum	Median level	Low	qRT-PCR	M	OS,DFS	7

*M, Multivariate analysis; U, Univariate analysis; DFS, Disease-free survival; OS, Overall survival; RFS, Recurrence-free survival; PFS, Progression-free survival; NR, Not reported; high, high expression; low, low expression*.

**Table 2:** Prognostic value of the microRNAs expression profile mentioned in the literature.

**microRNA**	**HR (95% CI)**	**Heterogeneity test**		**Sample size**	**Expression**	**Outcome**	**Number of microRNA**
		***I*^**2**^ (%)**	**Chi^**2**^ (*P*)**				
miR-133b	5.53 (2.58–11.83)	0.0%	0.939	100	Low expression	OS/DFS	2
miR-206	5.66 (2.69–11.88)	0.0%	0.914	100	Low expression	OS/DFS	2
miR-133b/206	9.48 (4.59–19.57)	0.0%	0.953	100	Low expression	OS/DFS	2
miR-195	4.23 (2.31–7.73)	0.0%	0.568	166	Low expression	OS/DFS	2
miR-152	0.13 (0.02–0.70)	–	–	80	Low expression	OS	1
miR-223	4.59 (1.84–11.45)	–	–	112	Low expression	OS	1
miR-326	3.90 (1.13–12.53)	–	–	60	Low expression	OS	1
miR-95-3p	4.22 (2.31–8.07)	–	–	133	Low expression	OS	1
miR-497	3.96 (2.39–6.58)	0.0%	0.868	185	Low expression	OS	2
miR-491	3.06 (1.56–6.00)	0.0%	0.928	102	Low expression	OS	2
miR-124	3.73 (2.27–6.12)	0.0%	0.841	114	Low expression	OS/DFS	2
miR-491-5p	2.68 (1.66–4.32)	0.0%	0.951	72	Low expression	DFS/OS	2
miR-101	4.16 (2.80–6.19)	0.0%	0.995	152	Low expression	OS/RFS	4
miR-139-5p	3.03 (2.17–4.23)	0.0%	0.707	98	Low expression	OS	2
miR-29a	5.68 (2.50–12.92)	0.0%	0.903	80	High expression	OS/DFS	2
miR-29b	5.71 (2.58–12.67)	0.0%	0.904	80	High expression	OS/DFS	2
miR-196a	6.59 (3.08–14.11)	0.0%	0.896	100	High expression	OS/DFS	2
miR-196b	6.64 (3.09–14.24)	0.0%	0.900	100	High expression	OS/DFS	2
miR-196a/196b	9.99 (4.82–20.69)	0.0%	0.979	100	High expression	OS/DFS	2
miR-221	7.26 (3.29–16.04)	0.0%	0.886	108	High expression	RFS/OS	2
miR-27a	3.36 (1.90–5.95)	0.0%	0.851	166	High expression	OS/DFS	2
miR-191	3.05 (1.75–5.31)	0.0%	0.593	100	High expression	OS/DFS	2
miR-300	5.07 (3.41–7.54)	0.0%	0.958	114	High expression	OS/DFS	4
miR-542-3p	7.83 (5.41–11.34)	0.0%	0.431	76	High expression	OS/PFS	4
miR-194	4.01 (2.53–6.36)	0.0%	0.695	124	Low expression	OS/DFS	2

*DFS, Disease-free survival; OS, Overall survival; RFS, Recurrence-free survival; PFS, Progression-free survival; –, Not available*.

### Prognostic Accuracy and Subgroup Analyses

**Figure 2:**
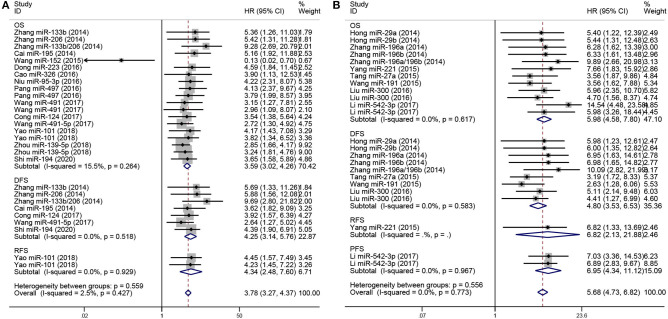
Forest plot for miRNA expression and prognosis in osteosarcoma, stratified by outcomes included (OS, DFS, RFS, PFS). **(A)** Low-expression miRNAs correlated with poor prognosis in osteosarcoma, stratified by outcomes (OS, DFS, RFS). **(B)** High-expression miRNAs correlated with poor prognosis in osteosarcoma, stratified by event times (OS, DFS, RFS, PFS).

To analyze the prognostic value of miRNA expression in osteosarcoma, forest plots of data from the 19 studies, in accordance with HRs and their 95% CIs, are shown in Figure 2. The HRs were calculated on the basis of low-expression or high-expression miRNAs, respectively. HR >1 or <1 implied poor or good prognosis for patients with osteosarcoma, respectively. The pooled HRs for low- and high-expression miRNAs were 3.78 (95% CI 3.27–4.37, *P* < 0.05) and 5.68 (95% CI 4.73–6.82, *P* < 0.05), respectively, and both tended to be associated with a poorer outcome. Additionally, low-expression miRNAs were stratified by outcomes, including OS (pooled HR = 3.59, 95% CI 3.02–4.26, *P* < 0.05), DFS (pooled HR = 4.25, 95% CI 3.14–5.76, *P* < 0.05), and RFS (pooled HR = 4.34, 95% CI 2.48–7.60, *P* < 0.05). Furthermore, high-expression miRNAs were classified by outcomes, including OS (pooled HR = 5.98, 95% CI 4.58–7.80, *P* < 0.05), DFS (pooled HR = 4.80, 95% CI 3.53–6.53, *P* < 0.05), RFS (pooled HR = 6.82, 95% CI 2.13–21.88, *P* < 0.05), and PFS (pooled HR = 6.95, 95% CI 4.34–11.12, *P* < 0.05), and these miRNAs were also associated with poor prognosis in osteosarcoma.

**Figure 3:**
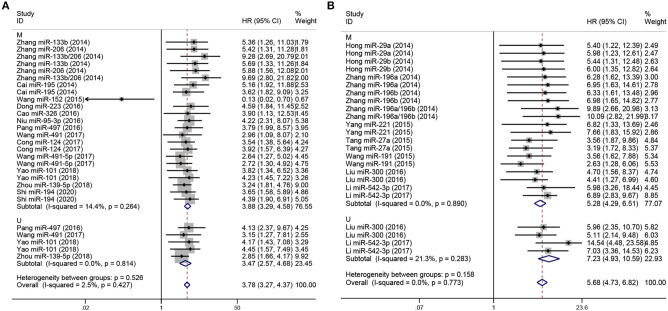
Forest plot for miRNA expression and prognosis of osteosarcoma, stratified by analysis method. **(A)** Low-expression miRNAs correlated with poor prognosis in osteosarcoma, stratified by analysis method (M and U). **(B)** High-expression miRNAs with poor prognosis in osteosarcoma, stratified by analysis method (M and U). The *p*-values for heterogeneity of HR by subgroup, and overall, are shown. M, Multivariate analysis; U, Univariate analysis.

**Figure 4:**
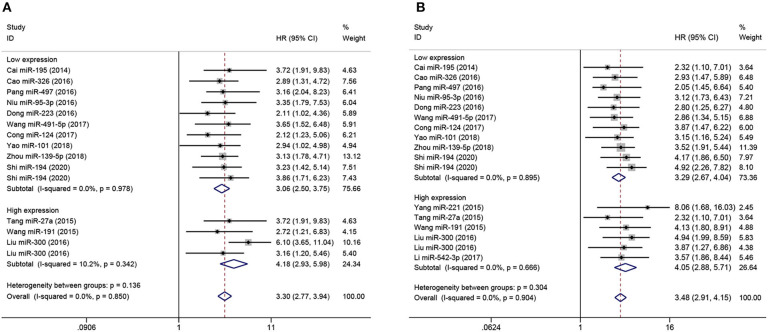
Forest plot of the correlation between metastasis, clinical stage, and miRNA expression in studies with poor overall survival. **(A)** Correlation between miRNA level and tumor metastasis in osteosarcoma with poor overall survival, stratified by miRNAs expression (high-expression miRNA or low-expression miRNA). **(B)** Correlation between miRNA level and clinical stage in osteosarcoma with poor overall survival, stratified by miRNA expression (high-expression miRNA or low-expression miRNA).

Subgroup analyses were performed according to analysis method and clinicopathological features in order to explore the correlation of miRNA expression on prognosis in osteosarcoma, as shown in Figures 3, 4, respectively. As shown in Figure 3A, subgroup analyses reveal that low expression levels of miRNA were significantly correlated with poor prognosis in osteosarcoma according to both multivariate (pooled HR = 3.88, 95% CI 3.29–4.58, *P* < 0.05) and univariate analyses (pooled HR = 3.47, 95% CI 2.57–4.68, *P* < 0.05). Similar results are shown in Figure 3B: multivariate (pooled HR = 5.28, 95% CI 4.29–6.51, *P* < 0.05) and univariate analyses (pooled HR = 7.23, 95% CI 4.93–10.59, *P* < 0.05) both indicate that high expression of miRNA are correlated with poor prognosis in osteosarcoma. Additionally, as shown in Figure 4, the level of expression of serum miRNAs is closely correlated with distant metastasis (pooled HR = 3.30, 95% CI 2.77–3.94, *P* < 0.05), and clinical stage (pooled HR = 3.48, 95% CI 2.91–4.15, *P* < 0.05) in osteosarcoma.

### Publication Bias and Sensitivity Analysis

**Figure 5:**
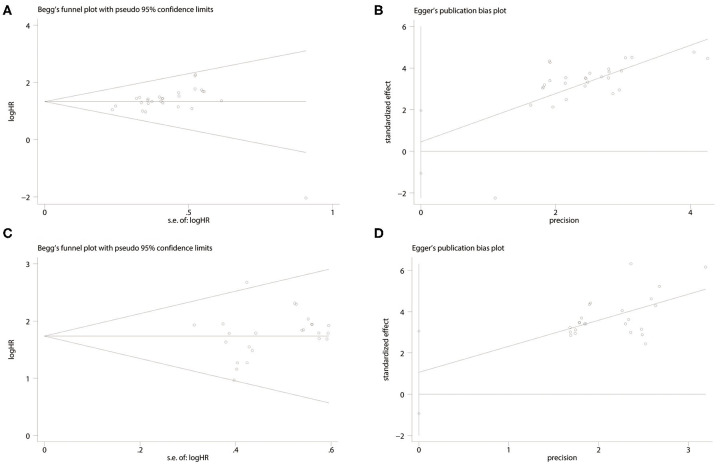
Forest plot of the publication bias. **(A)** Begg's funnel plot of publication bias for the association between miRNA low expression and poor prognosis. **(B)** Egger's test of publication bias for the association between miRNA low expression and poor prognosis. **(C)** Begg's funnel plot of publication bias for the association between miRNA high expression and poor prognosis. **(D)** Egger's test of publication bias for the association between miRNA high expression and poor prognosis.

The *P*-values for Begg's tests of low-expression miRNAs and high-expression miRNAs were 0.028 and 0.602, respectively, and the corresponding *P*-values for Egger's tests were 0.544 and 0.283. Furthermore, the funnel plots of Begg's and Egger's are all symmetrical demonstrating that there is no significant publication bias in this research (Figure 5). Sensitivity analyses revealed that none of the studies were outliers, suggesting that the pooled results of this research are credible.

## Discussion

Although osteosarcoma is the common malignant bone tumor (Kansara et al., 2014) and extensive progress has been made in the development of effective therapies (Bielack et al., 2002; Collins et al., 2013), patient prognosis remains unsatisfactory. Therefore, for improved treatment, management, and patient prognosis in osteosarcoma, the identification of novel prognostic biomarkers is critical. The potential utility of circulating miRNAs as non-invasive biomarkers has been demonstrated in several types of cancers (Zhou et al., 2016; Tan et al., 2019). Furthermore, blood testing is more easily accepted by patients than other invasive tests. Therefore, we conducted a meta-analysis of studies investigating the prognostic capacity of serum miRNAs in osteosarcoma. A more detailed subgroup analysis was also conducted to further examine the association between analysis methods, clinical stage, metastasis, and miRNA expression level in osteosarcoma.

We investigated 20 studies on 23 different miRNAs in osteosarcoma in this meta-analysis; these included 9 highly expressed miRNAs (miRNA-29a, miRNA-29b, miRNA-196a, miRNA-196b, miRNA-221, miRNA-27a, miRNA-191, miRNA-542-3p, and miRNA-300) (Hong et al., 2014; Zhang et al., 2014a; Tang et al., 2015; Wang T. et al., 2015; Yang et al., 2015; Li et al., 2018), and 14 miRNAs with low expression (miRNA-195, miRNA-223, miRNA-497, miRNA-491, miRNA-124, miRNA-101, miRNA-139-5p, miRNA-326, miRNA-133b, miRNA-206, miRNA-152, miRNA-95-3p, and miRNA-491-5p, miR-194) (Cai et al., 2015; Cao et al., 2016; Dong et al., 2016; Pang et al., 2016; Wang S. N. et al., 2017; Cong et al., 2018; Yao et al., 2018; Zhou et al., 2018; Shi et al., 2020). A previous meta-analysis has reported that aberrant expression of miRNAs, in terms of both elevated and downregulated expression, are associated with poor prognosis in osteosarcoma (Cheng et al., 2017). Similar to the above findings, our data confirm the observation that serum miRNAs with aberrantly elevated or downregulated levels of expression are strongly correlated with poor prognosis for osteosarcoma. Among osteosarcoma patients, high expression of serum miRNAs (miRNA-196a and miRNA-196b) and combined expression of miRNA-196a/miRNA-196b were independent prognostic factors for OS and DFS (Zhang et al., 2014a). Further, Frampton et al. (2014) reported that miRNA-21, miRNA-23a, and miRNA-27a are highly expressed in pancreatic tumor, and their combination could serve as a prognostic biomarker in this tumor. The above results suggest that the development of an optimal panel of miRNA expression would be useful biomarker to improve prognosis in osteosarcoma.

The levels of miRNA-27a, miRNA-191, miRNA-195, miRNA-497, miRNA-223, miRNA-124, miRNA-101, miRNA-139-5p, miRNA-326, miRNA-95-3p, miRNA-491-5p, and miRNA-300, miRNA-194 (Cai et al., 2015; Tang et al., 2015; Wang T. et al., 2015; Cao et al., 2016; Dong et al., 2016; Liu et al., 2016; Niu et al., 2016; Pang et al., 2016; Wang Z. et al., 2017; Cong et al., 2018; Yao et al., 2018; Zhou et al., 2018; Shi et al., 2020) are potentially associated with clinical stage as well as the tumor metastasis in osteosarcoma. miRNA-27a, miRNA-191, and miRNA-300 are highly expressed in osteosarcoma, while miRNA-195, miRNA-497, miRNA-223, miRNA-124, miRNA-101, miRNA-139-5p, miRNA-326, miRNA-95-3p, miRNA-194, and miRNA-491-5p are expressed at low levels. In particular, the high expression of miR-191 may affect cancer progression through various pathways, and is associated with therapeutic outcomes or poor prognosis in cancers (Elyakim et al., 2010; Li et al., 2017). As such, miR-27a and miR-191 serve as regulatory factors in osteosarcoma. Low expression of miR-195 may act as a regulator in hepatocellular carcinoma cells, with potential utility in cancer therapy (Xu et al., 2009). In addition, low expression of serum miR-497 may be associated with tumor development (Kong et al., 2015). miR-124 was found to be expressed at low levels and correlated with invasion and metastasis in hepatocellular carcinoma (HCC), which may predict poor prognosis (Zheng et al., 2012). Low expression of miR-101 has been shown to inhibit the progression of bladder transitional cell carcinoma, and may serve as a tumor suppressor gene in this disease (Friedman et al., 2009). miR-139-5p has been shown to be expressed at low levels, and is considered to act as a regulator of cell proliferation, metastasis, apoptosis, and the cell cycle (Zhang et al., 2014c). In glioblastomas, miR-326 shows low expression and acts as a tumor suppressor (Nawaz et al., 2016). These results demonstrate, at a molecular level, that miRNAs have diagnostic or prognostic utility that could be extended to other tumors. miR-195, miR-497, miR-223, miR-124, miR-101, miR-139-5p, and miR-326 act as tumor suppressors in cancers, which is consistent with the results obtained in our study. Therefore, the conclusions that high-expression or low-expression miRNAs are potential novel biomarkers for predicting prognosis in osteosarcoma are reliable.

However, this study has some limitations. All relevant publications may not have been included in the databases, and specific subgroup analyses showed mild heterogeneity. HRs and RRs were merged into HRs in the included literature, potentially leading to slight logical errors, finally the included studies' population limited to Chinese. Despite these limitations, this study suggests that miRNAs have potential utility as novel prognostic markers in osteosarcoma. Further investigation of the dynamic expressional profile of miRNAs during the entire course of the development and treatment of osteosarcoma is needed for clinical implementation of miRNAs as biomarkers in either diagnosis or prognosis.

Despite these limitations, the results of this study suggest that serum miRNAs represent excellent biomarkers of prognosis in osteosarcoma. We conclude that miRNAs in this systematic review with aberrantly low or high expression are indicative of poor prognosis in osteosarcoma. Further, the expression of miRNAs(miRNA-195, miRNA-27a, miRNA-191, miRNA-300, miRNA-326, miRNA-497, miRNA-95-3p, miRNA-223, miRNA-491-5p, miRNA-124, miRNA-101, miRNA-139-5p, miRNA-194) is correlated with clinical stage and tumor metastasis in this disease. However, the implementation of miRNAs as biomarkers for monitoring cancer progression, clinical stage and distant metastasis and guiding therapeutic interventions improving poor prognosis. Future studies should aim to address these critical aspects.

## Data Availability Statement

The raw data supporting the conclusions of this article will be made available by the authors, without undue reservation.

## Author Contributions

HL designed the study and collected data. HL and PW drafted the manuscript. PW, HY, JS, LD, XW, and CS contributed to the writing. JZ and JL contributed to the writing and review of the manuscript. All authors contributed to the article and approved the submitted version.

## Conflict of Interest

The authors declare that the research was conducted in the absence of any commercial or financial relationships that could be construed as a potential conflict of interest.
