# Supplementary material for: Serum-Derived microRNAs as Prognostic Biomarkers in Osteosarcoma: A Meta-Analysis
Source: Front Genet. 2020 Aug 11;11:789. doi: 10.3389/fgene.2020.00789 (PMC7431663; doi:10.3389/fgene.2020.00789)
Supplement: Supplementary file 1 [file Data_Sheet_1.zip › Supplementary materials/code.pdf]

- gen InLL=log(LL)
- gen InUL=log(UL)
- gen InHR=log(HR)
- gen logHR=ln(HR)
- gen selogHR=(ln(_UCI)-ln(_LCI))/3.92
